# Postoperative Effects of Somatosensory Foot Training on Balance and Walking Ability in Proximal Femoral Fractures: A Single-case Design Analysis

**DOI:** 10.1298/ptr.25-E10349

**Published:** 2025-09-11

**Authors:** Tomoki HAKAMATA, Junichi SUGANUMA, Kazuhiro CHIDORI

**Affiliations:** 1 Department of Rehabilitation, Kasai Central Hospital, Japan; 2 Department of Physical Therapy, Faculty of Nursing and Rehabilitation, Chubu Gakuin University, Japan

**Keywords:** Somatosensory training, Single-case design, Proximal femoral fractures, Timed-Up-and-Go test, Tau-U

## Abstract

**Objectives:**

By using a single-case design, we aimed to assess the impact of postoperative somatosensory foot training on walking balance and speed compared with conventional physical therapy interventions in 3 patients with proximal femoral fractures.

**Methods:**

This study included 3 patients in their 70s who sustained left proximal femoral fractures due to falls and underwent open reduction and internal fixation or hemiarthroplasty. An AB-type single-case design was employed, with phase A consisting of conventional physical therapy interventions and phase B involving additional somatosensory training. Each phase lasted for 6 days. The outcome measures included the Timed Up-and-Go (TUG) test and 10-m maximum walking speed. To determine interventional effects, effect sizes were calculated using the percentage of nonoverlapping data (PND) and the Tau-U coefficient.

**Results:**

Our PND analysis indicated a large effect size for the TUG and 10-m maximum walking speed outcome measures in all cases. Additionally, the Tau-U analysis showed that all patients exhibited significantly shorter TUG times and higher 10-m maximum walking speeds in phase B compared with phase A (p <0.05).

**Conclusions:**

Postoperative somatosensory foot training may be effective in improving walking balance and speed in patients with proximal femoral fractures.

## Introduction

Proximal femoral fractures are among the leading causes of hospitalization in older adults, often resulting from falls and are likely to require surgical intervention^1)^. Patients who undergo surgery for proximal femoral fractures often experience physical impairments such as decreased walking speed^2)^, impaired balance^3)^, reduced ability to perform activities of daily living^4)^, and muscle weakness^5)^. Decreased walking ability was observed in approximately 50% of the patients 12 and 24 months after surgery^6)^. A decrease in walking speed has been associated with an increased risk of falls^7)^ and may lead to a decline in balance ability^3)^. Additionally, patients with impaired balance are at higher risk of falls, which may lead to re-hospitalization^8)^. For these reasons, assessing the postoperative improvement in walking balance and walking abilities in patients who sustain proximal femoral fractures from falls and undergo surgery is an important issue in preventing subsequent falls.

Human gait and balance adjustments are integrated by the central nervous system based on afferent information from somatosensory inputs^9)^. Notably, it has been clarified that afferent sensory information from the feet and soles plays an important role in balance regulation^10)^. In this context, studies have shown that somatosensory training of the feet, which enhances the contribution of somatosensation and promotes perceptual learning, is beneficial for improving balance ability^11)^. In the study by Park, older adults with a history of falls were divided into interventional and control groups. Although the amount of sensory input from the soles was approximately the same for both groups, only the interventional group, which identified the hardness of the sponges placed on the soles, showed an improvement in balance ability as the number of errors in hardness discrimination decreased. Therefore, it is important to increase sensory input and appropriately discriminate somatosensory information from the feet and soles, thereby enhancing perceptual accuracy.

Coordinating ankle muscle activity and ensuring efficient functional treatment strategies for the ankle are essential for regulating gait and balance. Previous studies have suggested that incorporating comprehensive tasks, including proprioception of the ankle joint, in addition to identifying a single sensory modality, such as sponge hardness (pressure sensation), may improve balance function estimation, such as the Timed Up-and-Go (TUG) test^11)^ and walking speed^12)^. Furthermore, a previous study showed that somatosensory training improved 2-point discrimination (TPD) ability in the soles^13)^. Based on these previous studies, we hypothesized that intensive postoperative somatosensory training, including comprehensive tasks targeting the feet, may improve balance and walking ability in patients who have undergone surgery for proximal femoral fractures and have reduced walking ability.

In this study, we used a single-case design to investigate whether postoperative somatosensory training targeting the feet was more beneficial for improving balance ability and walking speed compared with standard physical therapy interventions in 3 patients with proximal femoral fractures following a fall. Additionally, we examined whether improvements in balance and walking ability were accompanied by an increase in the sensitivity of the TPD threshold at the soles

## Methods

### Participants

#### Case 1

A man in his 70s was diagnosed with a left femoral trochanteric fracture and underwent open reduction and internal fixation with a gamma nail. The study period comprised 12 days, from postoperative days 11 to 28. At the initial assessment, muscle strength, evaluated using manual muscle testing (MMT), was grade 4 in both the upper and lower limbs. The patient was able to walk independently within the ward using a T-cane. The Functional Reach Test (FRT) value was 12 cm, the TUG test result was 14.9 s, and the maximum walking speed in the 10-m walk test (10MWT) was 0.67 m/s.

#### Case 2

A woman in her 70s was diagnosed with a left femoral trochanteric fracture and underwent open reduction and internal fixation with a gamma nail. The study period covered 12 days, from postoperative days 7 to 22. The MMT score was grade 4 in both the upper and lower limbs. The patient was able to walk independently within the ward using a T-cane. The FRT value was 14 cm, the TUG test result was 16.8 s, and the maximum walking speed in the 10MWT was 0.64 m/s.

#### Case 3

A woman in her 70s was diagnosed with a left femoral neck fracture and underwent femoral head replacement. The study period covered 12 days, from postoperative days 9 to 25. The MMT score was grade 4 in both the upper and lower limbs. The patient was able to walk independently within the ward using a T-cane. The FRT value was 12 cm, the TUG test result was 14.1 s, and the maximum walking speed in the 10 MWT was 0.69 m/s. Cognitive function was assessed using Hasegawa’s Dementia Scale-Revised, and all cases scored 30/30.

### Study design

The study employed an AB-type single-case design. During phase A, which served as the baseline period, only conventional physical therapy was provided. In phase B, the interventional phase, somatosensory training was added to the conventional physical therapy. The duration of intervention was 80 min per session in phase A, consisting solely of conventional physical therapy, whereas in phase B, 40 min were allocated to conventional physical therapy and an additional 40 min to somatosensory training. The duration of each phase was 6 days.

### Outcome measures

The outcome measures included the TUG test, the 10 MWT maximum walking speed, the TPD threshold, and the FRT. The TUG test was used as an indicator of gait and balance ability^14)^. The test was performed once in a right-turn direction and once in a left-turn direction using a stopwatch, and the mean value of the 2 trials was calculated. The maximum walking speed was assessed using the 10 MWT, where the time required to walk 10 m was measured with a stopwatch, and the mean value of 2 trials was recorded^15)^. The TPD threshold was used as an indicator of spatial resolution on the soles^16)^. Measurements were conducted on the left hallux using a digital caliper (Shinwa, Niigata, Japan) while the patient was barefoot and in a supine position on a bed. The test was performed 3 times at the same distance, and the minimum distance at which at least 2 correct responses were obtained was defined as the TPD threshold^17)^. The mean value of 2 trials was used as the representative value. In this study, a reduction in the TPD threshold was defined as an improvement. The FRT value was used as an indicator of dynamic balance ability^18)^, and the mean value of 2 trials was calculated. Measurements were recorded after each intervention session in both phases A and B.

One therapist conducted both the assessments and interventions. The study content and phases were not blinded. The therapist, with 10 years of clinical experience in this area, had participated in both domestic and international training programs on somatosensory training. The therapist was responsible for both phases A and B.

### Intervention methods for each phase

In phase A, the conventional physical therapy intervention consisted of joint range-of-motion exercises, strength training and maintenance, stretching, standing and walking practice, and movement guidance. The content of the conventional physical therapy intervention in phase B was the same.

**Fig. 1.**
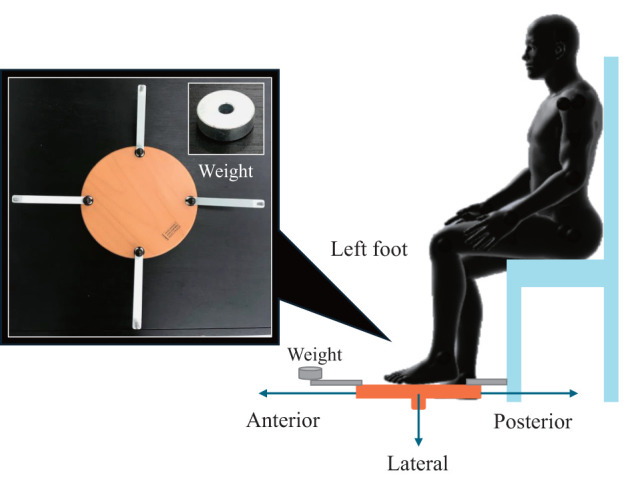
Discrimination task on the foot: Example of a task to discriminate a weight placed on the front-left side Weights were randomly placed at 4 positions (front, back, left, and right) on an unstable board, and participants were asked to identify the position of the weight. During the task, participants closed their eyes and performed automatic movements to discriminate the position of the placed weight. The foot used for the task was the operated side, and the weights used were 200 g, 100 g, or no weight. During the task, feedback was provided: correct responses were verbally confirmed, while incorrect responses led to a temporary interruption of the task, during which participants were instructed to open their eyes and visually check the position of the weight.

In phase B, based on previous studies, somatosensory training was conducted by placing the soles of the feet on a multi-axis unstable board (Cognitive Kit Set A2-2 series, Fumagalli, Ponte Lambro, Como, Italy) while the patient remained seated, and the patient was asked to discriminate between the positions of weights (Fig. 1)^13)^. The unstable board had weights randomly placed at 4 positions (front, back, left, right), and the patient was required to identify the location of the weights. During this task, the patient closed his or her eyes and performed automatic movements to discriminate between the locations of the placed weights. The foot used for the task was the surgical side, and the weights had a mass of 200, 100, or 0 g. Initially, a low-difficulty 1st task using a 200 g weight was conducted, and after 5 consecutive correct trials, the participant moved on to the 2nd task using a 100 g weight. The 2nd task also concluded after 5 consecutive correct trials. The 3rd task involved a combination of 200, 100, and 0 g weights, which were randomly used from the 3 types of weights. Feedback for the patients during the task included verbal confirmation for correct answers and a pause in the task for incorrect answers, with instructions to open their eyes and visually confirm the weight position. Through these tasks, the aim was for the patients to properly discriminate somatosensory information from the foot, encourage coordination of muscle activity around the foot, and facilitate effective functional treatment strategies for the ankle.

### Statistical analysis

The statistical analysis focused on the TUG test, 10 MWT maximum walking speed, TPD threshold, and FRT value. To assess the impact of the intervention, the standard deviation (SD) band method was used for each measure. The mean value ±2 SD for phase A (baseline) was calculated, and the number of phase B data points outside this range was counted^19)^. The effect size was calculated using the percentage of nonoverlapping data (PND)^20)^. Based on previous research, PND values of <50% were considered to have no effect, 50% ≤ PND <70% were deemed to have a questionable effect, 70% or more were considered effective, and 90% or more were considered very effective^21)^. Another measure of effect size, Tau-U^22)^, was also calculated. Tau-U values range from −1 to 1, and trends from phases A to B were calculated with reference to baseline trends. Based on previous research criteria, Tau-U values less than 0.20 were considered to have a small effect, 0.20–0.60 were deemed to have a medium effect, 0.60–0.80 were considered to have a large effect, and values exceeding 0.80 were deemed to have a very large effect^23)^. Statistical analysis was performed using R Studio version 2024.04.2(R Studio, Boston, MA, USA), and the statistical significance level was set at 5%.

### Ethical considerations

This study was conducted with approval from the Ethics Committee of Chubu Gakuin University, Seki, Japan (Ethical Approval Number: C24-0006). Participants were provided with a thorough explanation of the purpose of the study both orally and in writing beforehand, and their informed consent was obtained prior to participation on a voluntary basis.

## Results

**Fig. 2.**
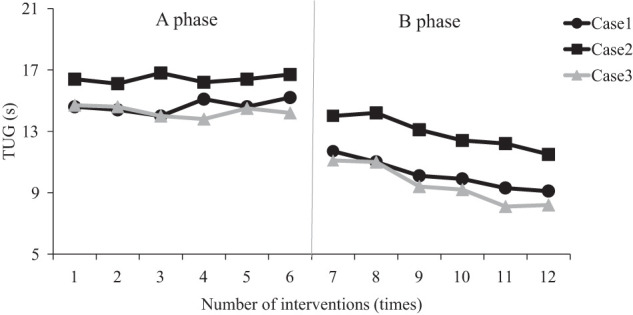
TUG duration for each case TUG, Timed Up and Go test

**Fig. 3.**
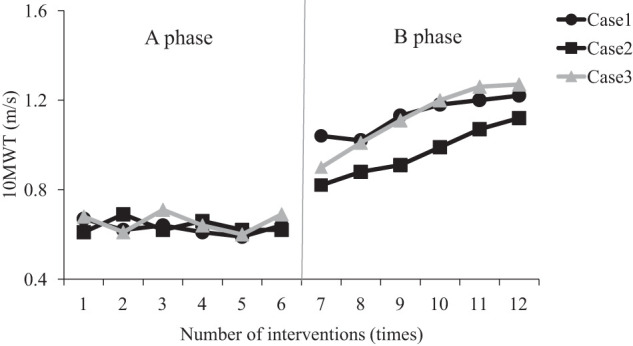
10 MWT maximum walking speed in each case 10MWT, 10-m walk test

The results of the TUG test and 10 MWT maximum walking speed for each patient are shown in Figs. 2 and 3. The mean ± SD values of each measure are shown in Table 1. The results of the SD range method for each evaluation item revealed that, in all cases, the phase B data exceeded the range of the phase A mean ± 2 SD. The PND was 100% for the TUG test, 10 MWT maximum walking speed, FRT, and TPD threshold in all cases, indicating a large effect size. The Tau-U coefficient for the TUG test showed that, in all cases, phase B was significantly shorter than phase A (Table 2). For patients 1 and 2, a significantly large effect size was observed, while patient 3 displayed a large effect size. Furthermore, in all cases, significant improvements were observed in 10MWT maximum walking speed, FRT, and TPD threshold values for phase B compared with phase A, with results exceeding 0.8, indicating a significantly large effect size that was consistent with prior studies.

**Table 1.** Mean values and standard deviations of the evaluations in each case

	Case 1		Case 2		Case 3	
	A phase	B phase	A phase	B phase	A phase	B phase
TUG (s)	14.6 (0.4)	10.1 (1.0)	16.4 (0.2)	12.9 (1.0)	14.3 (0.3)	9.5 (1.3)
10MWT (m/s)	0.6 (0.02)	1.1 (0.08)	0.6 (0.03)	0.9 (0.1)	0.6 (0.04)	1.1 (0.1)
FRT (cm)	12.5 (0.5)	23 (3.1)	13.6 (0.5)	21.8 (3.3)	11.8 (0.4)	21.1 (3.3)
TPD (mm)	23.8 (0.4)	12.6 (2.3)	24.3 (0.5)	14.8 (3.3)	25.6 (0.5)	15.1 (4.0)

The mean value of each evaluation item is presented, with the standard deviation shown in parentheses.

TUG, Timed Up and Go test; 10MWT, 10 meter walk test; FRT, Functional Reach Test; TPD, two-point discrimination

**Table 2.** Results of Tau-U in each case

Tau-U	Case 1		Case 2		Case 3	
	Trend A	A vs. B with A*	Trend A	A vs. B with A*	Trend A	A vs. B with A*
TUG	−0.4	1	−0.26	1	0.46	0.8
p	n.s.	p <0.01	n.s.	p <0.01	n.s.	p = 0.02
10MWT	−0.4	1	0	1	−0.06	1
p	n.s.	p <0.01	n.s.	p <0.01	n.s.	p <0.01
FRT	0.2	0.91	−0.26	1	−0.06	1
p	n.s	p <0.01	n.s.	p <0.01	n.s.	p <0.01
TPD	−0.2	1	0.13	0.94	0.13	0.94
p	n.s.	p <0.01	n.s.	p <0.01	n.s.	p <0.01

*Considering the baseline trend, we used A vs. B with trend. Tau-U is a metric used to assess the effect size of interventions in single-case designs, with values ranging from −1 to 1. An improvement of 0.20 may be considered a small change, 0.20–0.60 a moderate change, 0.60–0.80 a large change, and above 0.80 a large to very large change, depending on the context.

p, p value; n.s., not significant; TUG, Timed Up and Go test; 10MWT, 10 meter walk test; FRT, Functional Reach Test; TPD, two-point discrimination

The changes in the TUG test values from the start to the end of phase A were within the range of the minimum detectable change (MDC) value of 1.62 s reported in previous studies^24)^, while phase B results exceeded this range. Additionally, the changes in 10 MWT maximum walking speed values in phase A were within the range of 0.1 m/s, the MDC value reported previously^25)^, and exceeded this range in phase B.

## Discussion

Using a single-case study design, we investigated whether postoperative somatosensory training, in comparison with conventional physical therapy interventions, was beneficial for improving balance and walking abilities in 3 patients with proximal femoral fractures and a reduced walking capacity. Additionally, we examined whether improvements in balance and walking abilities were associated with improvements in the TPD threshold on the soles of the feet. Our results revealed that postoperative somatosensory training led to a reduced TUG test value, an elevated 10 MWT maximum walking speed, a longer FRT distance, and an improvement in the TPD threshold in all 3 patients (Table 2). These findings support the notion that the postoperative somatosensory training implemented in this study may be effective in improving balance and walking abilities, and that the improvement in TPD threshold may contribute to these improvements.

Previous studies on somatosensory training of the feet in older adults have shown that proprioceptive information, such as ankle joint kinesthesia, is related to balance and walking abilities^26)^. The present study found that older adults with poor balance had a decreased sense of ankle movement, which affected their walking speed. Based on this, the importance of postoperative somatosensory training of the feet has been suggested to improve balance and walking abilities. A study involving older adults who had previously experienced falls reported that the interventional group who underwent training to identify hardness on the soles showed a reduced TUG test value and a decrease in the center of pressure sway^11)^. In a study by Morioka et al., an intervention requiring older adults to identify sponge hardness had a significant impact on standing balance, with the interventional group showing a significant reduction in center of pressure sway and an increase in the FRT distance^27)^. A theoretical explanation for these interventional effects suggests that proprioceptive information from the body, including internal feedback, may be integrated, potentially promoting learning processes in the central nervous system^9)^. In this study, the repetitive performance of tasks that required the identification of kinesthetic information from the feet probably helped the patients to better discriminate somatosensory information from the feet and soles, leading to improvements in the TUG test, 10 MWT maximum walking speed, and FRT results.

Further, it has been suggested that the improvement in TPD threshold resulting from somatosensory training leads to enhanced balance abilities, as reflected in the TUG test and FRT measurements. In a study by Matsuno et al.^28)^, somatosensory training using a balance pad effectively improved the TPD threshold and enhanced balance abilities during static standing in older adults. In addition, a negative correlation between the TPD threshold and single-leg stance time has been reported^29)^. Melzer et al.^30)^ pointed out that an improved TPD threshold may lead to an inability to sense a center of pressure sway, potentially causing a decline in balance abilities. Based on these findings, it was suggested that an improvement in the TPD threshold through the identification of kinesthetic information from the feet enhances the perception accuracy of somatosensory information from the soles, enabling a better control of the center of mass of the body, which in turn improves balance abilities. Previous studies have reported that the TPD threshold increases with aging^29)^ and that older adults who have experienced falls have higher TPD thresholds than non-falling older adults^30)^. Based on these previous studies, it was inferred that the elevated TPD threshold observed in all cases was due to both aging and the fall history of the patients.

In all cases, the TUG test and 10 MWT maximum walking speed values exceeded the MDC values reported in previous studies during phase B^24,25)^, suggesting a favorable outcome. The MDC is an indicator that shows the magnitude of error in repeated measurements, such as retesting. Therefore, a change that exceeds the MDC value is considered an actual change that surpasses the measurement error in repeated testing^31)^. The MDC value for the TUG test has been reported as 1.62 s^24)^, and that for the maximum walking speed in a 10-m walk was 0.1 m/s^25)^. In the present study, since all patients showed values exceeding the MDC for both the TUG test and the maximum walking speed during phase B, it is highly likely that the intervention had an effect.

The present study has 4 limitations. First, the sample size was small, with only 3 cases included. To generalize the effects of postoperative somatosensory training, it is necessary to increase the number of cases and conduct a comparative study between an intervention group and a control group. Second, the experimental design presents limitations. In the AB design employed in this study, the timing of phases A and B differed, making it difficult to completely eliminate the influence of natural recovery processes, such as the postoperative disease stage and the phase of tissue healing. Because this design does not allow for a clear estimation of the causal relationship between the intervention and outcomes, future studies may need to adopt designs such as an ABAB design or a multiple baseline design to strengthen causal inferences. Third, because the same therapist conducted both the assessments and interventions, the possibility of measurement and intervention bias cannot be entirely excluded. Fourth, the sustainability of the intervention effects remains unclear. Future research should examine whether the effects of the intervention are maintained over time.

## Conclusions

In this study, we used a single-case study design to assess whether somatosensory training was beneficial for improving balance and walking speed compared with conventional physical therapy interventions in 3 patients with reduced walking ability after proximal femoral fractures. Additionally, we assessed whether improvements in balance and walking ability were accompanied by an increase in the TPD threshold at the soles. The results showed that the TUG test value decreased, 10 MWT maximum walking speed increased, FRT distance increased, and the TPD threshold improved. Based on these findings, we conclude that the somatosensory training performed in these cases may be effective in improving walking speed and balance, with improvements in the TPD threshold potentially contributing to these changes.
